# Antibacterial Natural Halimanes: Potential Source of Novel Antibiofilm Agents

**DOI:** 10.3390/molecules25071707

**Published:** 2020-04-08

**Authors:** Ignacio E. Tobal, Alejandro M. Roncero, Rosalina F. Moro, David Díez, Isidro S. Marcos

**Affiliations:** Departamento de Química Orgánica, Facultad de Ciencias Químicas, University of Salamanca, Plaza de los Caídos 1-5, 37008 Salamanca, Spain; ignaciotobal@usal.es (I.E.T.); alexmaron@usal.es (A.M.R.); rfm@usal.es (R.F.M.); ddm@usal.es (D.D.)

**Keywords:** halimanes, bacteria, marine origin, biofilm, diterpenes, tuberculosinol, isotuberculosinol, *ent*-halimic acid, synthesis

## Abstract

The development of new agents against bacteria is an urgent necessity for human beings. The structured colony of bacterial cells, called the biofilm, is used to defend themselves from biocide attacks. For this reason, it is necessary to know their structures, develop new agents to eliminate them and to develop new procedures that allow an early diagnosis, by using biomarkers. Among natural products, some derivatives of diterpenes with halimane skeleton show antibacterial activity. Some halimanes have been isolated from marine organisms, structurally related with halimanes isolated from *Mycobacterium tuberculosis.* These halimanes are being evaluated as virulence factors and as tuberculosis biomarkers, this disease being one of the major causes of mortality and morbidity. In this work, the antibacterial halimanes will be reviewed, with their structural characteristics, activities, sources and the synthesis known until now.

## 1. Introduction

A bacterial biofilm is a structured colony of bacterial cells that appears when the planktonic cells are encrusted in a polymeric matrix composed by exopolysacharides, proteins and nucleic acids made by themselves, and adhered to a live or inert surface [1]. In order to form part of a biofilm, the bacteria make important changes in their structures and metabolism. Recent studies have allowed researchers to identify the genetic expression of the biofilms, which it is quite different to planktonic cells. The bacterial biofilm facilitates the survival of the pathogenic bacteria in their biological ambient, protecting the bacteria from the immunity response of the host and of biocides such as antibiotics or disinfectants. The impact of biofilms on health is out for discussion, as biofilm formation increases bacterial resistance against antibiotics. The modern concept of biofilms was introduced by Costerton and co-workers [1]. Later on, some articles come out referring to the mycobacterial biofilm environment, but it was not until 1990s when the current concept appeared for biofilm [2]. New biofilm definitions have successively appeared, such as microbial-derived sessile community, characteristics of certain microorganisms that adhered to the surfaces forming complex microbial communities that live, interact and work in different ecosystems [3].

A biofilm can comprise approximately 15% of cells and 85% of the extracellular matrix. The latter is formed by exopolysacharides that form channels where water, enzymes, nutrients and residues circulate [3,4]. There, cells establish relationships and dependences, living cooperatively and communicating amongst themselves through chemical signals (quorum perception), regulating the gene expression in a different manner depending on the location inside the community, such as a tissue in a multicellular organism. As more than 60% of bacterial infections are caused by biofilms and are considered a clinical threat because of their growth in catheters or surgery instruments, these bacterial structures have raised great interest in study.

As biofilms can act as reservoirs of infectious agents, it is necessary to develop more protocols for disinfection, not only in clinical settings but also in animal farms and in food processing [2,5].

Tuberculosis, mainly caused by *Mycobacterium tuberculosis (M. tuberculosis)*, is an important source of morbidity and mortality worldwide, with almost two million deaths annually [6]. Due to an increasing level of multidrug and drug resistance, new drugs against tuberculosis are necessary.

*M. tuberculosis* biofilms that were recently discovered open up a new area of research. The treatment of these infections is more effective when antibiotics are used in the first steps of the biofilm development [2]. It has been demonstrated that biofilm is involved in *M. tuberculosis* resistance to antituberculosis drugs, although it is not clear how these findings can be applied to tuberculosis treatment. It has been demonstrated that the bacteria death ratio increases when a combination of antibiotics and antibiofilm agents are employed [2].

The *M. tuberculosis* biofilm plays a central role in the process of caseous necrosis and cavity formation in lung tissue and can be found in clinical biomaterials. A major part of antimicrobial resistance is due to the presence of a bacterial biofilm, as it affords protection against antibiotics that are normally active against the same bacteria in the planktonic state [2]. Some treatment failure of biofilm-forming microorganisms is due to antibiotic, disinfectant, and germicide resistance. Clinical experience demonstrates that these biofilms should be eradicated prior to controlling the infection. Resistance could be explained by different mechanisms, permeability, metabolic states, activation of resistance genes or persisting cells [7,8,9].

**Figure 1:**
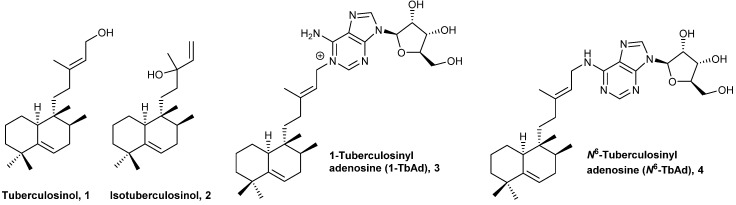
Tuberculosinols and derivatives.

For this reason, it is fundamental to develop new procedures that allow an early detection of the illness introducing new biomarkers. New therapies targeting the virulence factor (VF) formation are of maximum interest. VFs are involved in processes such as invasion, persistence, lysis and evasion of innate immune system responses, although VFs are not essential for bacterial growth outside the host cell. Two diterpenes tuberculosinol, **1**, and isotuberculosinol, **2**, can be considered VF in *M. tuberculosis* [6,10] (Figure 1).

In the biosynthesis of tuberculosinol, **1**, and isotuberculosinol, **2**, are involved two genes (Rv3377c and Rv3378c) found only in virulent species of genus *Mycobacterium* (such as *M. tuberculosis* and *M. bovis*). These genes could not be found in avirulent species of genus *Mycobacterium* (such as *M. smegmatis* and *M. avium*) [11,12], so they may be involved in the infection processes of these bacteria. The lower virulence of *M. bovis* in comparison with *M. tuberculosis* could be explained as these genes only seem to be functional in *M. tuberculosis* and not in *M. bovis* [13,14]. 

Tuberculosinol, **1**, and isotuberculosinol, **2**, roduced in vivo by *M. tuberculosis* [15,16] (in a 1:1 ratio), inhibit phagolysosome maturation as well as macrophage phagocytosis, plus a synergistic effect increased by the coexistence of both compounds. The pathogenicity of *M. tuberculosis* decreases the phagocytic capacity [11]. Thus, both tuberculosinol, **1**, and isotuberculosinol, **2**, biosynthetic proteins (Rv3377c and Rv3378c) are essential for the bacteria’s survival inside the macrophage [11,17,18]. Both enzymes are new potential targets for the development of novel drugs.

Two new natural tuberculosinol derivatives, with an adenosine unit at C15 of the diterpene, have been isolated from *M. tuberculosis* [19,20], 1-TbAd, **3**, and *N*^6^-TbAd, **4**, (Figure 1). It has been observed, in a comparative lipidomic assay between *M. tuberculosis* and *M. bovis* that these two diterpenes occur in higher amounts in *M. tuberculosis* and comprise >1% of all *M. tuberculosis* lipids, so they could serve as an abundant chemical marker of *M. tuberculosis* [19,21]. In addition, in this study it has been proved that the Rv3378c enzyme is responsible for 1-TbAd, **3**, formation; this protein appears to be a tuberculosinyl transferase (prenyl transferase).

In this manner, Rv3377c and Rv3378c are new targets for anti-infective therapies against tuberculosis that block virulence factor (tuberculosinols) formation [6]. Consequently, tuberculosis could be related to these halimane diterpenoids.

Tuberculosinol, **1**, isotuberculosinol, **2**, and analogues:1-tuberculosinyl adenosine (1-TbAd), **3**, and N^6^-tuberculosinyl adenosine (N^6^-TbAd), **4**, are halimanes that could be considered VF of *M. tuberculosis* and tuberculosis illness [18,19,20,22,23,24,25,26,27,28,29]. Nowadays, these compounds are being tested as biomakers for tuberculosis.

## 2. Halimanes of Marine and Bacterial Origin

**Figure 2:**
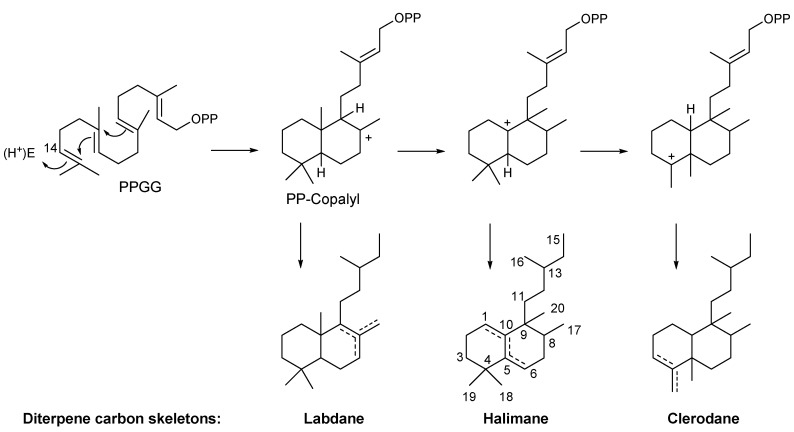
Biosynthesis of some bicyclic diterpenes.

Halimanes are members of the diterpene family that proceed from geranylgeranyl pyrophosphate and can be considered between labdane and clerodane diterpenes from a biogenetic point of view [22,30]. (Figure 2) Unlike labdane and clerodane diterpenes, more than a milliard compounds of each group are known, but barely 250 halimanes are known. 

**Figure 3:**
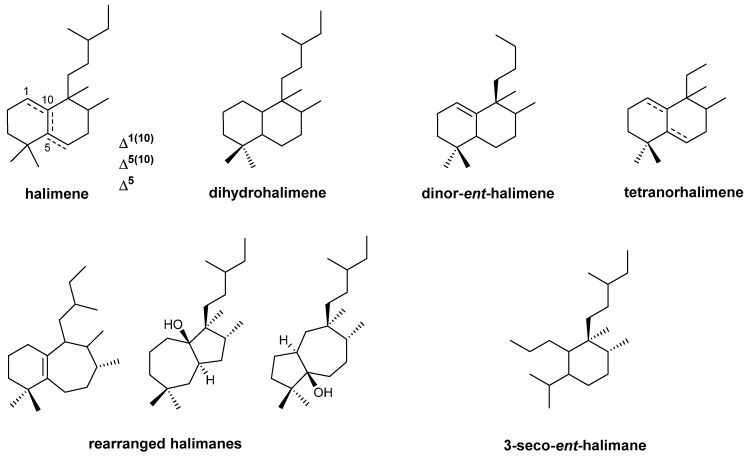
Some halimane skeletons.

Halimane diterpenes can be divided into different groups according to the annular double bond position. In this manner, halim-1(10)-ene, halim-5(10)-ene and halim-5-ene can be found (Figure 2). Among halimanes, other groups can also be found: dihydrohalimenes, rearranged halimanes, nor-halimanes and seco-halimanes. In Figure 3 appear some skeletons corresponding to these halimanes.

**Figure 4:**
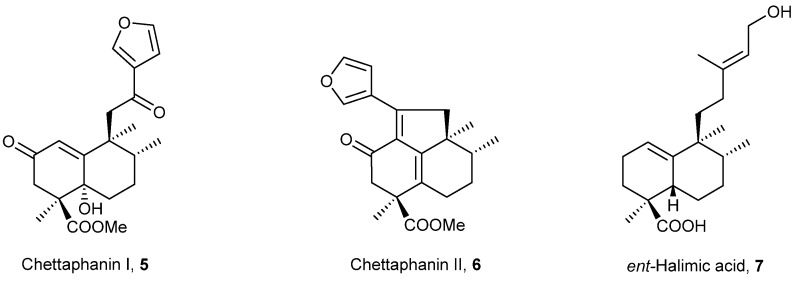
Some important halimanes.

In Figure 4, some of the most significative natural halimanes appear. Chettaphanin I, **5,** and chettaphanin II, **6**, **[23,24]** isolated from *Adenochlaena siamensis* are the first two halimanes known. Their structures were spectroscopically and X-ray determined in 1970, but their absolute configuration was not possible to be determined at that moment. *ent*-Halimic acid, **7**, and its acetylderivative [25,30], are the main components of *Halimium viscosum* (Villarino de los Aires), a plant of the Cistaceae family from which the majority of halimanes have been isolated. Due to this, this diterpene group is named after the gender of *Halimium viscosum* [22]. *ent*-Halimic acid, **7**, and its acetylderivative, were used as starting materials for the synthesis of chettaphanin I, **5**, and chettaphanin II, **6**, establishing in 2003 the absolute configuration for the mentioned natural products [31]. In addition to the halimanes described in this manuscript, there are a large number of very interesting halimanes because of other bioactivities as antitumoral, anti-inflammatory, antimicrobial, antifungal, germination inhibitors, etc [30].

## 3. Classification

In marine organisms and bacteria, halimanes are not very abundant, but they are very interesting due to their biological properties. These compounds have been isolated from mollusks have been isolated from mollusks (*Austrodoris, Spurilla*), sponges (*Agelas, Raspailia, Dysidea*), tunicates (*Cystodytes*), cnidaria (*Echinomuriceae,* medusa, anemonae, polyps or corals). Halimanes isolated from marine organisms have been divided into three groups, according to their structural characteristics: simple halimanes, halimane-glycerol and halimane-purines derivatives. In other groups, the halimanes with antibacterial activity that proceeds from bacteria and plants will be studied.

In this review, we have included not only halimanes found in marine organisms, but also halimanes isolated from bacteria and plants, because these halimane-purines are close structurally. These halimane-purines, as halimane derivatives of mixed biogenesis, follow similar biosynthetic routes to the ones that act as the enzyme Rv3378c.

### 3.1. Marine Simple Halimanes, **8–14**

**Figure 5:**
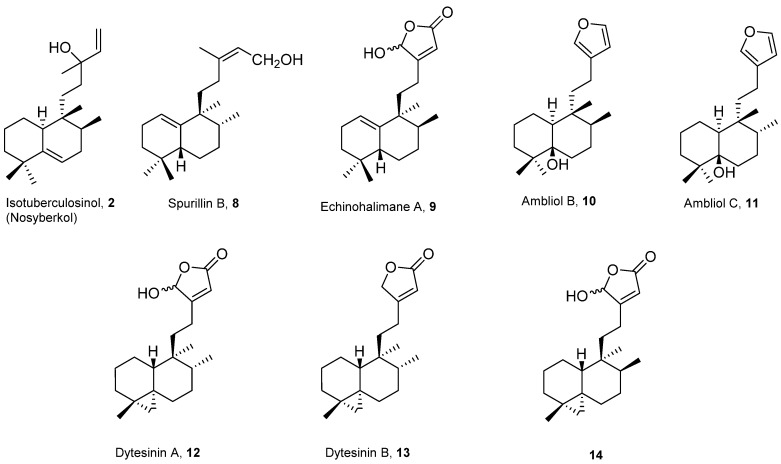
Halimanes of marine origin.

Among diterpene found in marine organisms (Figure 5) (Table 1), proper halimanes of different groups are found: halimanes **2**; *ent*-halimane, **8**; 8-epi-*ent*-halimane, **9**; two dihydrohalimenes, **10** and **11**; and three cyclopropylhalimanes **12**–**14**. Four of them present a butenolide or γ-hydroxibutenolide in the side chain, two are furanyl derivatives and spurillin B, **8**, that presents an unsaturated side chain with a cis double bond, which is unusual among the natural halimanes. Nosyberkol was isolated as a single stereoisomer in 2004 from the Nosy Be Islands (Madagascar) sponge *Raspailia sp* [32]. The C-13 configuration was not determined, and the absolute configuration remains undetermined. Some years later, nosyberkol was identified as isotuberculosinol, **2**, one of the metabolites isolated from *M. tuberculosis* that appears with tuberculosinol. The structures of the last compounds were corroborated by synthesis, establishing in this manner their absolute configuration as halimanes [29,33].

### 3.2. Halimane-Glycerol Derivatives Isolated from Marine Organisms: **15–18**

**Figure 6:**
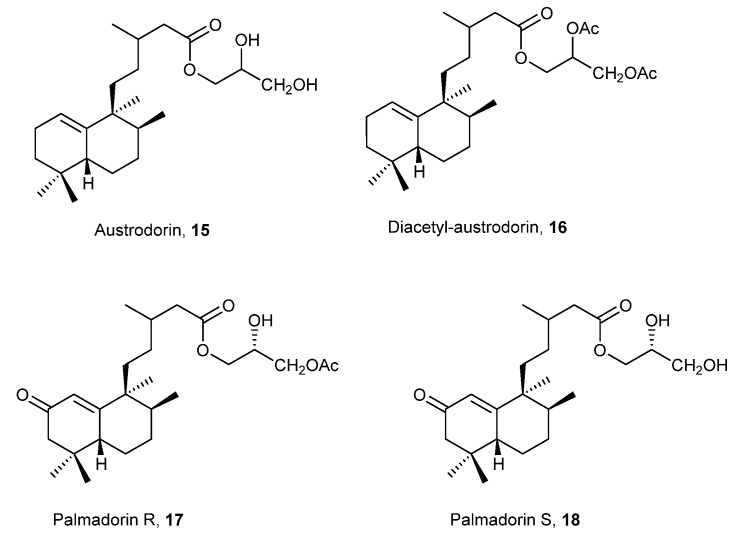
Halimane-glycerol derivatives.

A series of allomones was isolated from the Antarctic nudibranch, including diterpene glycerides, involved in the defense of those nudibranchs. All of them, **15**-**18**, are 8-epi-*ent*-halim-1(10)-ene glycerol esters, with the glycerol fragment free or esterified as acetates [40,41,42] (Figure 6) (Table 2).

### 3.3. Halimane-Purines Derivatives: **19–29**

**Figure 7:**
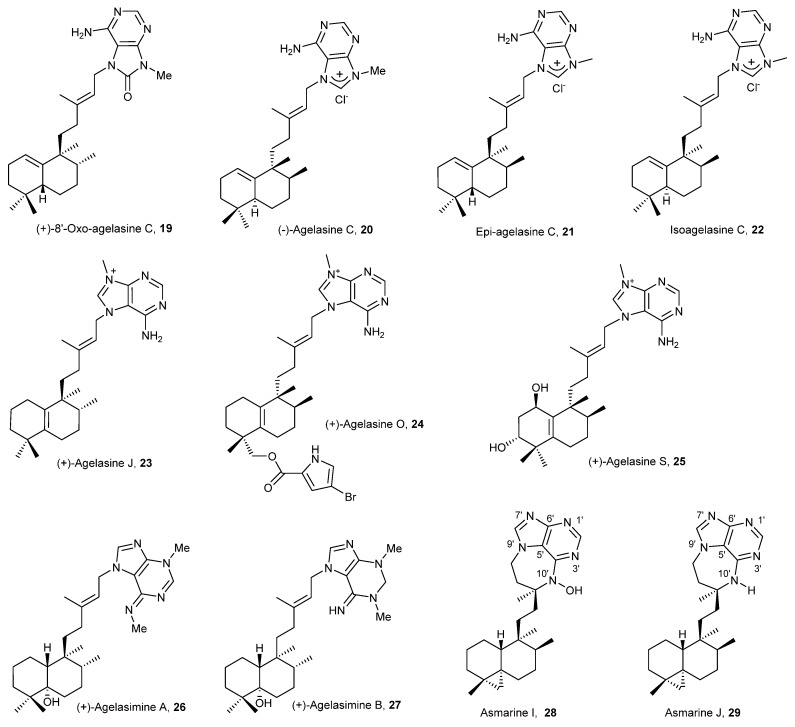
Halimane-purines.

Eleven halimane purines, mainly isolated from marine sponges, have been characterized. (Figure 7) (Table 3). Among them, the following halimane families can be found: *ent*-halim-1(10)-enes **19**, halim-1(10)-enes **20**, 8-epi-*ent*-halim-1(10)-enes **21**, **22**, *ent*-halim-5(10)-ene **23**, halim-5(10)-enes **24**, **25**, dihydro-*ent*-halimanes **26**, **27** and cyclopropyl-8-epi-*ent*-halimanes **28**, **29**. (Figure 7) All of them were isolated from sponges of the genus *Agellas,* except for asmarine I, **28**, and asmarine J, **29**, which were isolated from *Raspailia sp* [43]. These compounds show antibacterial, antifungal, antimalarial, and cytotoxic activities, inhibition of adenosine transfer into rabbit erythrocytes and Ca^2+^ channel antagonistic action and α1 adrenergic blockade, among others. Some of these compounds possess antifouling activity against macroalgae, and so can be useful in the fishing industry as an alternative to metals in anti-adherent mixtures. Antifouling substances with no or reduced toxicity should be discovered or developed [44]. Halimane-purines constitute very interesting natural products for further biological and chemical research owing to their biological activities [20]. Several of these compounds show antibacterial activity, and are structurally similar to the diterpene purines isolated from *M. tuberculosis* (1-TbAd, **3**, and *N^6^*-TbAd, **4**), although in these ones the purine appears glycosylated in a different union with the diterpene [44].

### 3.4. Halimanes Isolated from Bacteria

**Figure 8:**
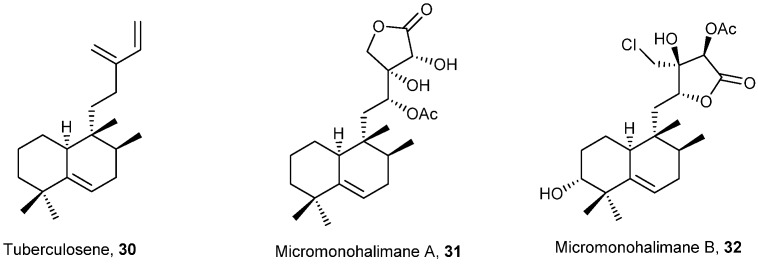
Some halimanes from bacteria

Compounds of this group (Figure 1 and Figure 8), (Table 4) have been isolated from bacteria, but nosyberkol (isotuberculosinol), **2**, has also been found in marine sponges of the genus *Raspailia sp*. All of them are halim-5-enes and characterized by their antibacterial activities.

Compounds tuberculosinol, **1**, and isotuberculosinol, **2**, are produced by *M. tuberculosis* [15,16]. Until now, the presence of **1** or **2** in the cultured cells of 12 nonpathogenic *Mycobacterium* species has not been detected [16]. It has been observed that tuberculosinol, **1**, and isotuberculosinol, **2**, (in a 1:1 ratio, with **2** being a mixture of the diastereomers 13*R*-isotuberculosinol (**2*R***) and 13*S*-isotuberculosinol (**2*S***) in a 1:3 ratio) inhibit phagolysosome maturation and macrophage phagocytosis in human-like cells [11].

A bacterial class II diterpene cyclase, DTC, (cyclase B type) that produces halima-5,13-dienyl diphosphate has been identified [27,55]. *M. tuberculosis* Rv3377c gene has been effectively identified, and the encoded DTC has been proved to be responsible for the production of the halimane skeleton.

Recently the structure of the diterpene tuberculosinol/isotuberculosinol synthase (Rv3378c) from *M. tuberculosis* has been reported [56]. The biosynthesis of tuberculosinols is catalyzed by two enzymes: Rv3377c, tuberculosinyl (halima-5,13-dien-15-yl) diphosphate synthase, and Rv3378c, tuberculosinol/(*R/S*)-isotuberculosinol synthase. Rv3377c is a DTC classified as class II that transforms GGPP into tuberculosinyl diphosphate (TPP) with a halimane core [23,57], while Rv3378c is a diterpene synthase that converts TPP into tuberculosinol, **1**, or (*R/S*)-isotuberculosinols, **2**, acting as a phosphatase/isomerase [11,17].

It is probable that the biosynthesis of the diterpene purines isolated from marine organisms follows a similar path to 1-TbAd, **3**, and *N^6^*-TbAd, **4**, in which enzyme homologs to Rv3378c could be involved, probably expressed in the genome of the surrounding microbiome around the macro organism. 

Tuberculosene, **30**, (Figure 8) has been obtained by enzymatic reaction from a mixture of GGPP with tuberculosinyl diphosphate synthase and CYC2 enzyme from the bacteria *Kitasatospora griseola* [28,58]. Micromonohalimanes A and B (**31** and **32**, from *Micromonospora* sp. [59]), which present antibacterial activity, have been characterized. Micromonohalimane B **32** is the only halimane which includes a chlorine atom in its structure.

Recently, new knowledge was reported about the evolution of the bacteria that causes tuberculosis in animals and human beings. To know how it can be differentiated, the different bacteria lines will increase the comprehension of bacteria origins that cause the illness and the genetic mechanisms involved. 

### 3.5. Antibacterial Halimanes Isolated from Plants

**Figure 9:**
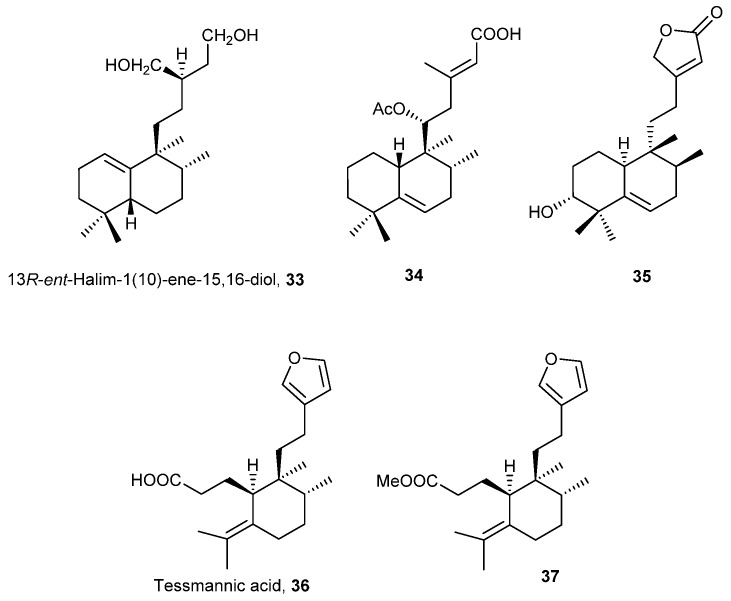
Antibacterial halimanes from plants

Antibacterial halimanes isolated from plants are shown in Figure 9 and Table 5. All of them are *ent*-halimanes, except for **35**, which is a halimane.

## 4. Halimane Synthesis

The following will describe the synthesis known until now of different halimanes isolated from marine organisms and bacteria. 

### 4.1. Halimane-Purine Synthesis. (+)-Agelasine C

**Scheme 1:**
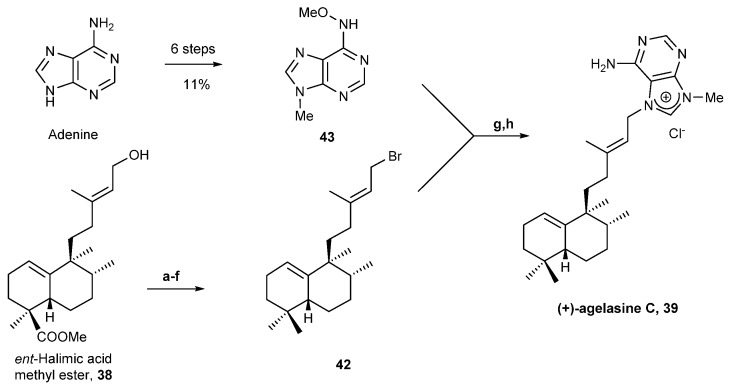
(**a**) DHP, *p*-TsOH, C_6_H_6_ (98%); (**b**) LAH, Et_2_O, 0 °C and then rt (99%); (**c**) TPAP, NMO (94%); (**d**) diethylene glycol, NH_2_NH_2_·H_2_O, KOH, 175–230 °C (81%); (**e**) *p*-TsOH, MeOH (81%); (**f**) CBr_4_, PPh_3_, DCM (76%); (**g**) DMA, 50 °C; (**h**) Zn, MeOH, H_2_O, AcOH (13% two steps).

**Figure 10:**
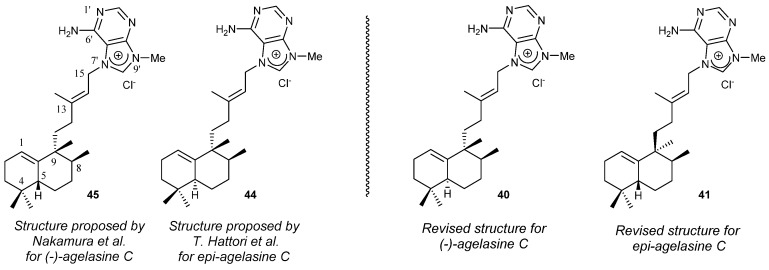
Structure proposed and revised for (−)-agelasine C and epi-agelasine C.

*ent*-Halimic acid methyl ester, **38**, has been used as a starting material for the synthesis of several terpene-alkaloids, such as (+)-agelasine C, **39**, (Scheme 1) [45,68,69,70,71,72]. With this synthesis, the authors were able to establish the structure and absolute configuration of the natural product (−)-agelasine C, **40**, correcting the structure originally proposed for epi-agelasine C, for this one was proposed the structure of **41 [45]**. (Figure 10)

Epi-agelasine C presents antifouling activity and in order to establish its absolute configuration it was designed with a synthesis using *ent*-halimic acid as starting material [45] (Scheme 1).

The synthesis of **42** (Scheme 1) was achieved in six steps from *ent*-halimic acid methyl ester, **38**. The sequence started with the reduction of the methoxycarbonyl group of C-18 to methyl, that has been carried out in other synthesis of biologically active compounds [73,74] and finally to install the bromoderivative of C-15. Coupling of **42** with adenine fragment **43** led in two steps to **39 [45]**.

The physical properties of **39** are very different to those of the natural product epi-agelasine C for which the structure **44** was proposed, which should be revised. However, ^1^H and ^13^C NMR of **39** are identical to those of (−)-agelasine C, for which the structure of **45** was originally proposed. In addition, the rotation of **39** and one of the natural products, (−)-agelasine C, are similar in absolute value but with the opposite sign. Accordingly, the natural product (−)-agelasine C has structure **40** (Figure 10) that it is the enantiomer of the synthesis product **39** (+)-agelaine C. By comparison of the spectra and rotations of **39** and those of the natural product epi-agelasine C, the structure of **41** for the natural product epi-agelasine C was proposed. (Figure 10)

### 4.2. Synthesis of (+)-agelasimine A, **26**, and (+)-agelasimine B, **27**

**Scheme 2:**
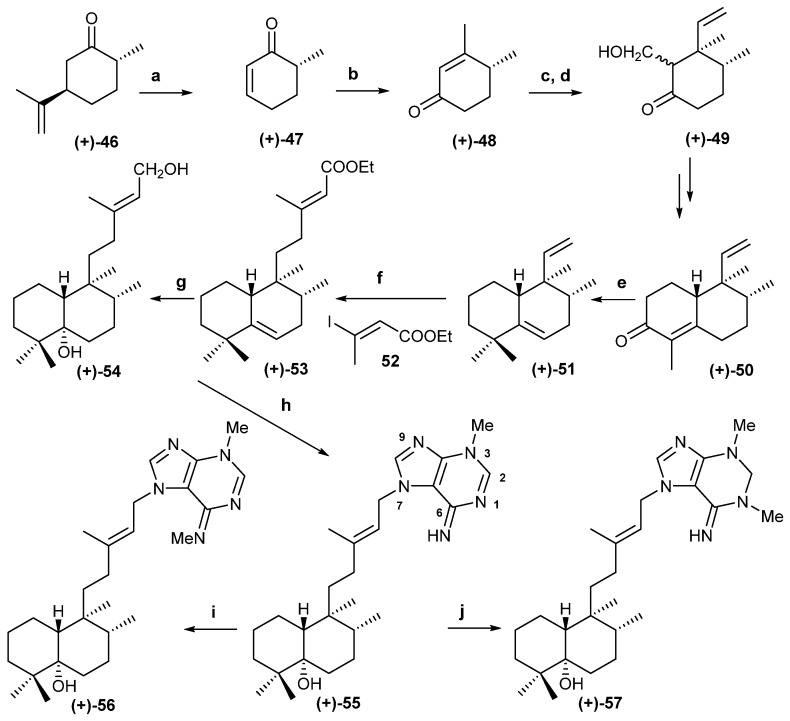
(**a**) 1) O_3_, MeOH, 2) FeSO_4_, Cu(OAc)_2_ (44%); (**b**) 1) MeLi, 2) PCC (94%); (**c**) 1) CH_2_=CHMgBr, CuBr·Me_2_S, Me_3_SiCl; (**d**) aq. HCHO, Yb(OTf)_3_ (75%); (**e**) 1) MeI, *t*-BuOK, *t*-BuOH, 2) NH_2_NH_2_.H_2_O, KOH, diethylene glycol 130 °C 1 h, 190 °C 3h (72%); (**f**) 1) 9-BBN, 2) **52**, PdCl_2_(dppf), CsCO_3_, Ph_3_As (67%); (**g**) 1) *m*-CPBA; 2) DIBAL, −78 °C; 3) LAH, THF reflux (51%); (**h**) 1) PBr_3_, 2) 3-methyladenine, AcNMe_2_; 3) aq. NaOH (60%); (**i**) 1) MeI, AcNMe_2_; 2) aq. NaOH(61%); (**j**) 1) NaBH_4_, 2) MeI, AcNMe_2_; 3) aq. NaOH (41%).

(+)-Agelasimine A, **26**, and (+)-Agelasimine B, **27**, are two diterpene-adenine derivatives isolated from the orange sponge *Agelas mauritiana* (Figure 7). The synthesis of **26** and **27** was carried out by Ohba and co-workers [53], starting with (+)-*trans*-dihydrocarvone, **46**, allowing them to establish the absolute configuration of both natural products. The authors previously developed a reaction sequence similar to that done for the racemic (±)-agelasimine A and (±)-agelasimine B [75,76]. The asymmetric synthesis of (+)-agelasimine A, **26**, and (+)-agelasimine B, **27**, was done from (+)-*trans*-dihydrocarvone, **46**, (Scheme 2) through enones **47** and **48** to access the key intermediate **49**, which was transformed into the bicyclic enone **50** by a procedure previously described. Transformation of **50** into the required diterpenic diol (+)-**54** was carried out by a seven-step sequence, among which Suzuki cross-coupling was necessary to complete the side chain. The synthesis of (+)-**55** was done starting from **54** in three steps: bromation, alkylation, with 3-methyladenine and neutralization of the hydrobromide salt. Methylation of **55** leads to compound (+)-**56** that was identical to the natural product (+)-agelasimine A. Reduction of **55** followed by methylation and neutralization leads to (+)-**57**, which is identical to the natural product (+)-agelasimine B. In this manner, the structures were corroborated and finally the absolute configuration of the natural products (+)-agelasimine A and (+)-agelasimine B was established.

### 4.3. Synthesis of Tuberculosinol, **1**, and Isotuberculosinol, **2**

The development of effective new drugs against bacteria and biofilms of *M. tuberculosis* is absolutely necessary and urgent. In this line, the development of new therapies for the inhibition of the virulence factor (VC) formation such as tuberculosinol, **1**, and isotuberculosinol, **2**, (13*R* and 13*S*) is of special interest, where these two halimanes show the main interest and projection. The structures of **1** and **2** have been confirmed by synthesis [29,33,77], enabling researchers to achieve a structural revision of the diterpenes isolated from *M. tuberculosis*, assigning to isotuberculosinol the same structure of nosyberkol, **2**, (isolated from *Raspailia sp*). 

### 4.4. Snider’s Synthesis of Tuberculosinol, **1**, and Isotuberculosinol, **2**

**Scheme 3:**
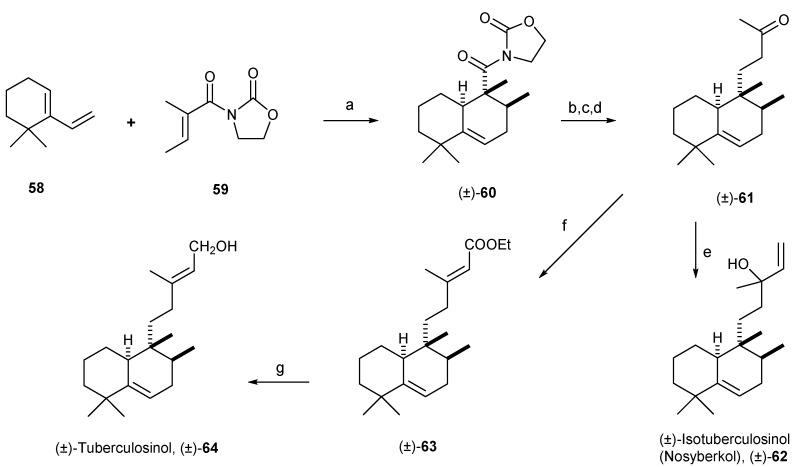
(**a**) Me_2_AlCl, CH_2_Cl_2_ (54%, 10:1 *exo/endo*); (**b**) 1) LiBH_4_, THF/H_2_O; 2) Dess-Martin periodinane, CH_2_Cl_2_ (77%); (**c**) acetone/MeOH, NaOMe (60%); (**d**) 1) Li, NH_3_, EtOH; 2) Jones oxidation (90%); (**e**) CH_2_=CHMgBr, THF (88%); (**f**) (EtO)_2_POCH_2_COOEt, NaH, THF (84%); (**g**) DIBAL, CH_2_Cl_2_ (92%).

For tuberculosinol, **1,** and isotuberculosinol, **2,** racemic synthesis, Snider and co-workers [33] used as a key step *exo*-cycloaddition (Scheme 3). Cycloaddition of **58** with *N*-tigloylisoxazolidinone, **59**, and Me_2_AlCl provides a mixture of the desired *exo*-Diels-Alder adduct **60** and the *endo* adduct (54%, 10:1 *exo/endo*). Reduction of (±)-**60**, Dess-Martin periodinane oxidation, followed by condensation of the aldehyde with acetone in the presence of NaOMe leads to an enone that by reduction with Li in NH_3_ gives the key intermediate (±)-**61**. The addition of vinylmagnesium bromide to methylketone (±)-**61** gives a diastereoisomer mixture (±)-**62** which has spectroscopic properties identical to the natural products nosyberkol and isotuberculosinol. The reaction of (±)-**61** with triethylphosphonoacetate leads to (±)-**63**, the reduction of which gives (±)-**64**, an identical product to the natural tuberculosinol [27].

### 4.5. Sorensen’s Synthesis of Tuberculosinol, 1, and Isotuberculosinol, **2**

**Scheme 4:**
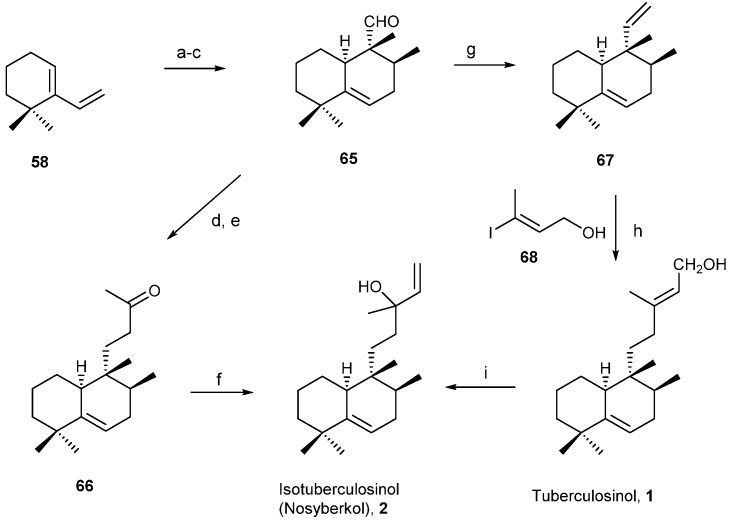
(**a**) Ethyl tiglate, neat, 160 °C, (71%) (2:1 *exo*/*endo*); (**b**) LAH, THF, (56%); (**c**) SO_3_·pyridine, NEt_3_, CH_2_Cl_2_-DMSO (86%); (**d**) acetone, NaHMDS, THF (87%); (**e**) 10 mol% Rh(PPh_3_)_3_Cl, HSiEt_3_, CH_2_Cl_2_ (83%); (**f**) vinylmagnesium bromide, THF (93%); (**g**) Ph_3_PCH_3_Br, KHMDS, THF (91%); (**h**) 9-BBN, THF; then 10 mol% PdCl_2_(dppf), Ph_3_As, CsCO_3_, **68**, DMF (73%); (**i**) 20 mol% CuCl_2_, acetone (20%).

The synthesis of tuberculosinol, **1,** and isotuberculosinol, **2,** carried out by Sorensen and co-workers [29], was used as key reaction in an *exo*-selective Diels-Alder reaction (Scheme 4). Cycloaddition of diene, **58**, with ethyl tiglate and ulterior reduction followed by chromatographic separation in supercritical conditions gave an enantioenriched material, that by oxidation gave **65**. Condensation of the last compound with acetone and reduction in the presence of Wilkinson´s catalyst gave methylketone, **66**. Vinylmagnesium bromide addition to ketone **66** gave the epimers mixture **2**, the spectroscopy of which was coincident with the natural products nosyberkol and isotuberculosinol.

Methylenation of **65** gave diene, **67**, that by hydroboration palladium-mediated cross-coupling with (*E*)-3-iodobut-2-en-1-ol **68** provides tuberculosinol, **1**. The reaction of **1** with catalytic copper(II) chloride led to isotuberculosinol (nosyberkol), **2**.

### 4.6. Barrero´s Synthesis of Isotuberculosinol, **2**

**Scheme 5:**
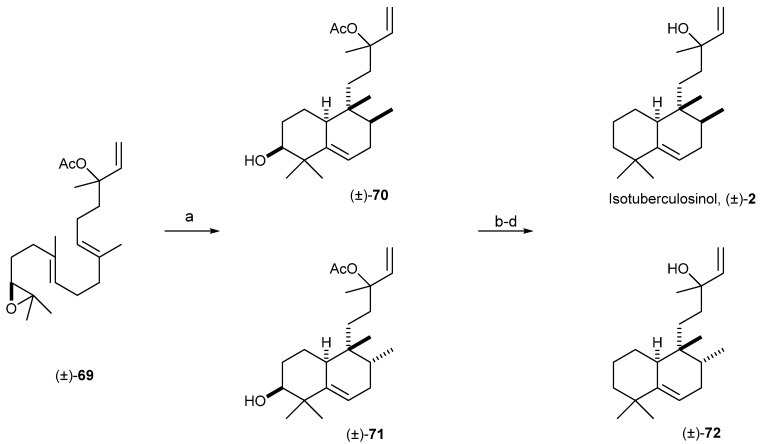
(**a**) Et_2_AlCl, (53%); (**b**) C_6_F_5_OCSCl; (**c**) Bu_3_SnH, AIBN; (**d**) LAH (65%, three steps).

Recently, a racemic, elegant and fast biomimetic synthesis of isotuberculosinol, (±)-**2**, based in the cascade cyclization and Lewis acid catalyzed rearrangement of epoxypolyprenes was described [77] (Scheme 5). Treatment of chiral geranyllinalool epoxide, (±)-**69**, with Et_2_ClAl in DCM at −78 °C gives a diasteroisomeric mixture (±)-**70**-(±)-**71** (53%, 1:2.5). Deoxygenation of each using the Barton–McCombie methodology and final reduction with LAH leads to isotuberculosinol, (±)-**2**, and 8-epi-isotuberculosinol, (±)-**72**, in global yields of 10% and 25%, respectively, from epoxy derivative, (±)-**69**.

### 4.7. Tuberculosinyl Adenosine (1-TbAd), **3**, and N^6^-Tuberculosinyl Adenosine (N^6^-TbAd), **4**, Syntheses

**Scheme 6:**
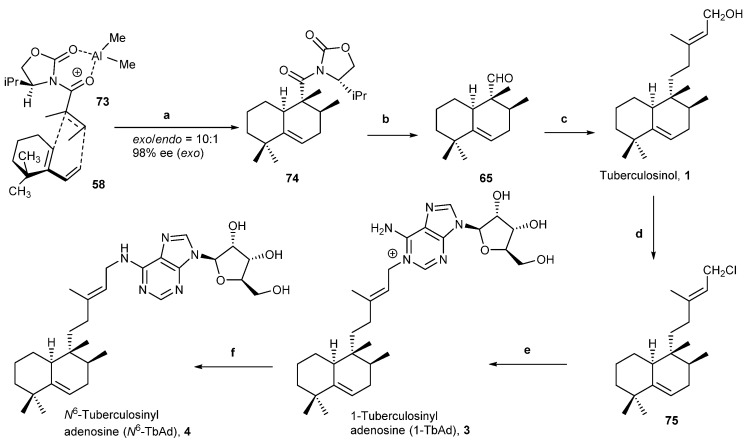
(**a**) Me_2_AlCl, CH_2_Cl_2_ (59%, 10:1 exo/endo); (**b**) 1) *n*-BuLi, EtSH, THF; 2) LAH, THF; 3) TPAP, NMO, CH_2_Cl_2_ (87%); (**c**) 1) NaHMDS, acetone, THF; 2) RhCl(PPh_3_)_3_, Et_3_SiH, CH_2_Cl_2_; 3) (EtO)_2_POCH_2_COOEt, NaH, THF; 4) DIBAL, CH_2_Cl_2_ (55%); (**d**) NCS, Me_2_S, CH_2_Cl_2_ (quantitative); (**e**) Adenosine, NaI, DMF, rt (76%); (**f**) Me_2_NH, H_2_O, rt (quantitative).

Nowadays tuberculosinol derivatives 1-tuberculosinyl adenosine (1-TbAd), **3**, and *N*^6^-tuberculosinyl adenosine (*N*^6^-TbAd), **4**, recently isolated [19,21] and characterized, are being assayed as specific biomarkers for *M. tuberculosis*. Biosynthetically *N*^6^-TbAd, **4**, could come from 1-TbAd, **3**, in vivo in *M. tuberculosis*, by a Dimroth rearrangement [19]. The synthesis of 1-TbAd, **3**, and *N*^6^-TbAd, **4**, has been carried out by 10- and 11-step sequences, respectively, using as a key step a chiral auxiliary-aided Diels-Alder reaction **73 [20]** (Scheme 6) that gives **74** (98% ee, 59%, 10:1 *exo/endo*). From **74** is obtained tuberculosinol, **1**, in seven steps. 1-TbAd, **3**, was obtained in two steps from **1** through the chloride derivative **75** and *N*^6^-TbAd, **4**, was achieved later from **3** by a Dimroth rearrangement.

## 5. Conclusions

The early attack against biofilm formation is fundamental for health in bacterial infections. For this reason, as tuberculosis constitutes one of the major mortality and morbidity causes, the introduction of new agents as biomarkers in this disease can be an important hallmark in the treatment of tuberculosis, especially the multiresistant varieties.

Most of the reviewed halimanes in this work show antibacterial activity and tuberculosinol, isotuberculosinol and their derivatives 1-TbAd, **3**, and *N^6^-*TbAd, **4** are considered as virulence factor (VF) in *M. tuberculosis*. Due to this, the proteins that participate in their biosynthesis (Rv3377c and Rv3378c) are new targets for anti-infective therapies against tuberculosis that block the virulence factor (tuberculosinols) formation. Thus, halimanes are very interesting tools for fighting against bacteria and biofilm communities. The introduction of new agents as tuberculosis biomakers can facilitate the treatment of this illness. 

## Figures and Tables

**Table 1:** Natural sources and activities of marine origin halimanes.

Marine Halimanes	Natural Sources	Activity	References
Nosyberkol, isotuberculosinol, **2**	*Raspailia sp.* *M. tuberculosis*		[29,32,33]
Spurillin B, **8**	*Spurilla sp.*		[34]
Echinohalimane A, **9**	*Echinomuricea sp*	Cytotoxic, neutrophil elastase inhibitor	[35,36]
Ambliol B, **10**	*Dysidea amblia*		[37,38]
Ambliol C, **11**	*D. amblia*		[38]
Dytesinin A, **12**	*Cystodytes sp.*		[39]
Dytesinin B, **13**	*Cystodytes sp.*		[39]
**14**	*Echinomuricea sp.*	Cytotoxic, anti-inflammatory	[39]

**Table 2:** Natural sources and activities of marine halimane-glycerols.

Marine Halimane-Glycerol Derivatives	Natural Sources	Activity	References
Austrodorin, **15**	*Austrodoris kerguelenensis* (Syn. of *Doris kerguelenensis*)	Self-defense	[40]
Diacetyl austrodorin, **16**	*A. kerguelenensis*	Self-defense	[40]
Palmadorin R, **17**	*A. kerguelenensis*		[41,42]
Palmadorin S, **18**	*A. kerguelenensis*		[41,42]

**Table 3:** Natural sources and activities of halimane-purines.

Halimane-Purines	Natural Sources	Activity	References
(+)-8’-Oxo-agelasine C, **19**	*Agelas mauritiana*		[43]
(−)-Agelasine C, **20**	*Agelas sp.* *A. citrina*	Inhibitory effects on Na,K-ATPase, antifungal, antimycobacterial	[45,46,47,48]
Epi-agelasine C, **21**	*Agelas sp.*	Antifouling, antimycobacterial	[45,48,49]
Isoagelasine C, **22**	*A. nakamurai*	Antifungal, antibacterial	[50]
(+)-Agelasine J, **23**	*A. mauritiana*	Antimalaria, antimicrobial, cytotoxic	[50,51]
(+)-Agelasine O, **24**	*Agelas sp.*	Antibacterial, antifungal	[52]
(+)-Agelasine S, **25**	*Agelas sp.*	Antibacterial, antifungal	[52]
(+)-Agelasimine A, **26**	*A. mauritiana*	Cytotoxic Adenosine transfer into rabbit erythrocytes inhibition. Ca^2+^-channel antagonistic action. α1 Adrenergic blockade	[53,54]
(+)-Agelasimine B, **27**	*A. mauritiana*	Cytotoxic Adenosine transfer into rabbit erythrocytes inhibition. Ca^2+^-channel antagonistic action. α1 Adrenergic blockade	[53,54]
Asmarine I, **28**	*Raspailia sp.*	Cytotoxic	[32]
Asmarine J, **29**	*Raspailia sp.*	Cytotoxic	[32]

**Table 4:** Natural sources, activities of halimanes from bacteria.

Bacterial Halimanes	Natural Sources	Activity	References
Tuberculosinol, **1**	*M. tuberculosis*		[12,14,17,18,26,27,28,29,33]
Nosyberkol, Isotuberculosinol, **2**	*Raspailia sp.* *M. tuberculosis*		[17,26,27,28,29,32,33]
Tuberculosene, **30**	*Kitasatospora griseola*		[28,58]
Micromonohalimane A, **31**	*Micromonospora sp.*	Antibacterial	[59]
Micromonohalimane B, **32**	*Micromonospora sp.*	Antibacterial	[59]
1-Tuberculosinyl adenosine (1-TbAd), **3**	*M. tuberculosis*	*M. tuberculosis* biomarker	[19,20,60,61]
*N*^6^-Tuberculosinyl adenosine (*N*^6^-TbAd), **4**	*M. tuberculosis*	*M. tuberculosis* biomarker	[19,20,60]

**Table 5:** Natural sources, activities of antibacterial halimanes from plants

Antibacterial Plant Halimanes	Natural Sources	Activity	References
13*R*-*ent*-halim-1(10)-en-15,16-diol, **33**	*Vellozia kolbekii*	Antitumour, antimicrobial	[62]
11*R*-Acetoxy-*ent*-halima-5,13*E*-dien-15-oic acid, **34**	*Plectranthus ornatus*	Antimicrobial	[63,64,65]
**35**	*Colophospermum mopane*	Antimicrobial	[66]
Tessmannic acid, **36**	*Tessmannia* *densiflora*	Antibacterial, antifungal, mosquito repellent, weak mosquitocidal	[67]
Tessmannic acid methyl ester, **37**	*T. densiflora*	Antibacterial, antifungal, mosquito repellent, weak mosquitocidal	[67]
